# Synthesis, crystal structure and magnetic properties of poly[[diaqua{μ_6_-2-[bis­(carboxyl­atometh­yl)amino]­terephthalato}­dicobalt(II)] 1.6-hydrate]

**DOI:** 10.1107/S2056989021008355

**Published:** 2021-08-17

**Authors:** Jie Ma, Wen-Zhi Zhang, Yong Liu, Wen-Tao Yi

**Affiliations:** a College of Chemistry, Chemical Engineering and Materials Science, Zaozhuang University, Zaozhuang, Shandong, 277160, People’s Republic of China

**Keywords:** crystal structure, 2-aminodi­acetic terephthalic acid, tetra­nuclear Co^II^ units, layered structure

## Abstract

The asymmetric unit of the polymeric title compound {[Co_2_(C_12_H_7_NO_8_)(H_2_O)_2_]·1.6H_2_O}_*n*_ comprises two Co^II^ ions, which are coordinated by fully deprotonated 2-aminodi­acetic terephthalic acid (adtp^4–^) and terminal water mol­ecules in distorted octa­hedral N_1_O_5_ and O_6_ coordination environments. The title compound features tetra­nuclear Co^II^ units bridged by *κ* ^3^ *O*:*O*:*O*′- and *κ* ^3^ *O*:*O*,*O*′-carboxyl­ate groups, which are joined into ribbons *via syn–anti* carboxyl­ate bridges. The parallel adtp^4–^ ligands with an alternately reversed arrangement further link adjacent Co^II^ ribbons into (010) layers, which are assembled into a three-dimensional supra­molecular network *via* inter­molecular hydrogen bonds. The disordered water solvent mol­ecules are situated in channels parallel to [100]. Magnetic measurements and analyses reveal that the title compound displays anti­ferromagnetic behaviour. The purity of the title compound was characterized by X-ray powder diffraction.

## Abstract 2

The title compound, {[Co_2_(C_12_H_7_NO_8_)(H_2_O)_2_]·1.6H_2_O}_*n*_, features tetra­nuclear Co^II^ units bridged by *κ* ^3^ *O*:*O*:*O*′- and *κ* ^3^ *O*:*O*,*O*′-carboxyl­ate groups from deprotonated 2-aminodi­acetic terephthalic acid, which are joined into Co^II^ ribbons *via syn–anti* carboxyl­ate bridges. The parallel-aligned adtp^4−^ ligands with an alternately reversed arrangement further link adjacent Co^II^ ribbons into (010) layers, which are assembled into a three-dimensional supra­molecular architecture *via* inter­molecular hydrogen bonds.

## Chemical context   

Over the last two decades, coordination polymers (CPs) have become one of the most attractive fields in chemistry because of their fascinating structures and promising applications as solid functional materials in adsorption and separation (Gan *et al.*, 2020 ▸; Yang *et al.*, 2020 ▸; Qian *et al.*, 2020 ▸; Islamoglu *et al.*, 2020 ▸), catalysis (Bavykina *et al.*, 2020 ▸), sensing (Allendorf *et al.*, 2020 ▸), luminescence (Rice *et al.*, 2020 ▸) and magnetism (Thorarinsdottir & Harris, 2020 ▸; Wang *et al.*, 2019*b* ▸). Multi­carb­oxy­lic acids have been employed to synthesize compounds comprising of various dimensional structures such as chains, layers and three-dimensional frameworks. Immense efforts have been devoted to the construction of CPs for successful predictions and the rational design of definite structures; many significant advances in the construction of CPs have occurred by employing well-defined rigid multi­carb­oxy­lic acids (Padial *et al.*, 2020 ▸; Li *et al.*, 2020*b* ▸; Wang *et al.*, 2019*a* ▸; Shen *et al.*, 2017 ▸; Pang *et al.*, 2017 ▸). However, using semi-rigid or flexible ligands, predictions are still tricky and confusing owing to the diversity of ligand configurations, the formation of various polynuclear metal units and the influence of weak inter­atomic inter­actions.

Our previous studies have focused on the construction of CPs based on semi-rigid multi­carb­oxy­lic acids with the aminodi­acetate moiety such as 2-aminodi­acetic terephthalic acid (H_4_adtp) (Liu *et al.*, 2009 ▸). The *ortho*-carboxyl­ate group of H_4_adtp can be regarded as three carb­oxy­lic arms attached to one amino nitro­gen atom. The three arms can chelate and/or bridge metal ions through their carboxyl­ate groups into polynuclear metal units or chains. The residual phenyl carboxyl­ate group can cross-link the polynuclear metal units or chains into layers or three-dimensional frameworks. In previous work, we have reported the supra­molecular hydrogen-bonded pillar-layered structure of [Mn(H_2_adtp)(H_2_O)]_*n*_ where the three arms connect Mn^II^ ions into layers with Mn^II^ chains and H_2_adtp ligands joined by hydrogen bonding act as pillars (Ma *et al.*, 2015 ▸). Herein we report the layer structure of the title compound, {[Co_2_(C_12_H_7_NO_8_)(H_2_O)_2_]·1.6H_2_O}_*n*_ (**I**), based on fully deprotonated H_4_adtp as one of the ligands. The crystal structure, power X-ray diffraction pattern and magnetism of (**I**) were also studied in detail.
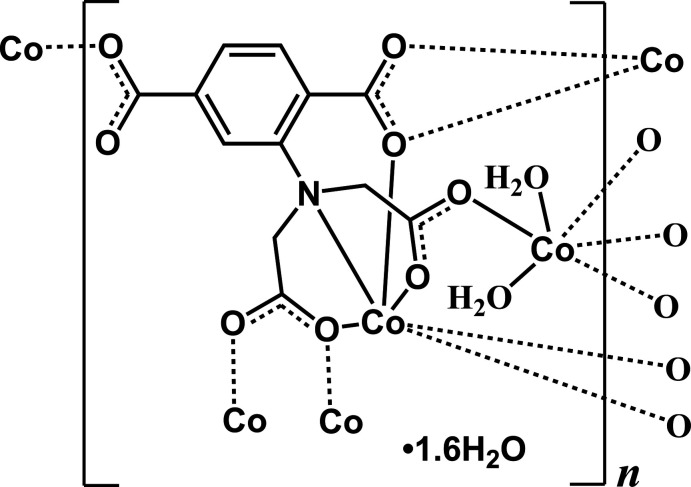


## Structural commentary   

**Figure 1:**
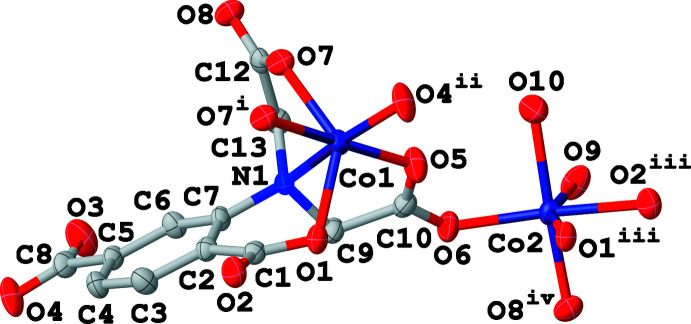
Coordination environments of the Co^II^ ions in (**I**) with displacement ellipsoids drawn at the 50% probability level; H atoms and the disordered lattice water molecules have been omitted for clarity. Symmetry codes refer to Table 1 ▸.

**Figure 2:**
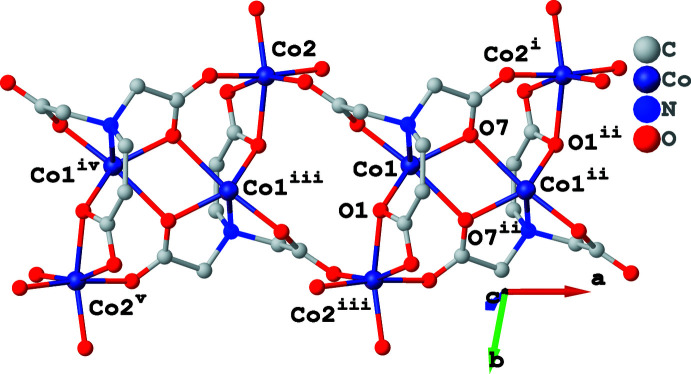
Tetra­nuclear Co^II^ units and a Co^II^ ribbon in (**I**). Phenyl and *meta*-positioned carboxyl­ate groups and the disordered lattice water molecules have been omitted for clarity. [Symmetry codes: (i) 1 + *x, y, z*; (ii) 1 − *x*, 1 − *y*, 1 − *z*; (iii) −*x*, 1 − *y*, 1 − *z*; (iv) −1 + *x*, *y*, *z*; (v) −1 − *x*, 1 − *y*, 1 − *z*.]

**Figure 3:**
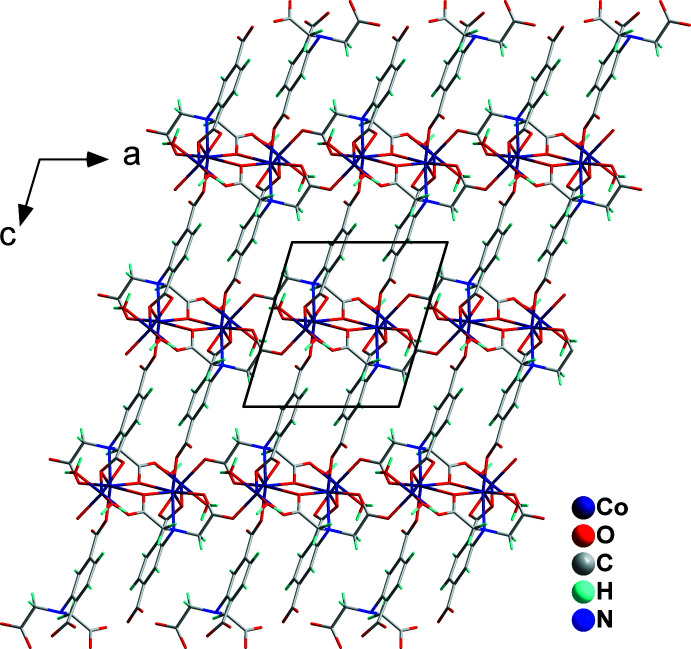
A view along [010], emphasizing the layered arrangement in the crystal structure of (**I**).

The asymmetric unit of (**I**) comprises two Co^II^ ions, one adtp^4–^ ligand, two terminal water ligands and 1.6 disordered solvent water mol­ecules. Regarding the adtp^4–^ ligand, one carboxyl­ate group (C12, O7, O8) of the aminodi­acetate moiety adopts a *κ* ^3^-*O*:*O*:*O*′ coordination mode and the other one (C10, O5, O6) employs a *syn*–*anti* bidentate bridging fashion, whereas the carboxyl­ate group in the *ortho*-position (C1, O1, O2) coordinates in a *κ* ^3^-*O*:*O*,*O*′ mode and that in the *meta*-position (C8, O3, O4) binds to one Co^II^ ion in monodentate fashion (see Scheme). As shown in Fig. 1 ▸, Co1 and Co2 are both six-coordinated and located in distorted octa­hedral environments with an N_1_O_5_ coordination set for Co1 and an O_6_ set for Co2. The adip^4–^ ligand chelates Co1 with the amino nitro­gen atom (N1) and carboxyl­ate oxygen atoms (O1, O5 and O7) from the aminodi­acetate moiety and its *ortho*-positioned carbox­ylate group. The residual *cis*-related sites are occupied by one *meta*-positioned carboxyl­ate oxygen atom (O4^ii^) and one aminodi­acetate oxygen atom (O7^i^) from two other adip^4–^ ligands (for symmetry codes refer to Table 1 ▸). The *ortho*-positioned carboxyl­ate group (O1^iii^ and O2^iii^) from another adip^4–^ ligand chelates Co2, two *cis*-related positions of which are occupied by two aminodi­acetate oxygen atoms (O8^iv^ and O6) from two different adip^4–^ ligands. The remaining two *cis*-related sites of Co2 are occupied by two terminal water ligands (O9 and O10). The length of the Co—N bond is 2.241 (3) Å and the Co—O distances are between 1.992 (3) and 2.362 (3) Å, which are all in the expected ranges. As shown in Fig. 2 ▸, two inversion-related adtp^4–^ ligands bridge two pairs of Co^II^ ions (Co1, Co1^ii^, Co2^i^ and Co2^iii^) into a tetra­nuclear unit with their *κ* ^3^ *O*:*O*:*O*′-carboxyl­ate groups from the aminodi­acetate moieties and *ortho*-positioned *κ* ^3^ *O*:*O*,*O*′-carboxyl­ate groups (Li *et al.*, 2020*a* ▸; Zhang *et al.*, 2019*a* ▸,*b* ▸; Liu *et al.*, 2018 ▸), wherein two equivalent *μ*_2_-oxygen atoms (O7 and O7^i^) from *κ* ^3^ *O*:*O*:*O*′-carboxyl­ate groups doubly bridge Co1 and Co1^ii^ into a dinuclear unit. The dinuclear unit is further joined with two equivalent Co2^i^ and Co2^iii^ atoms *via κ* ^3^ *O*:*O*:*O*′-carboxyl­ate groups and *μ*_2_-oxygen bridges (O1 and O1^i^) from *κ* ^3^ *O*:*O*,*O*′-carboxyl­ate groups. Adjacent tetra­nuclear units are linked into a ribbon *via* double *syn*–*anti* bridging carboxyl­ate groups from the aminodi­acetate moieties. The closest Co1⋯Co2 and Co1⋯Co1 distances in the ribbon are 3.7074 (8) and 3.5762 (8) Å, respectively. Parallel-aligned adtp^4–^ ligands with an alternately reversed arrangement bind adjacent Co^II^ ribbons into a layer extending parallel to (010) (Fig. 3 ▸).

## Supra­molecular features   

**Figure 4:**
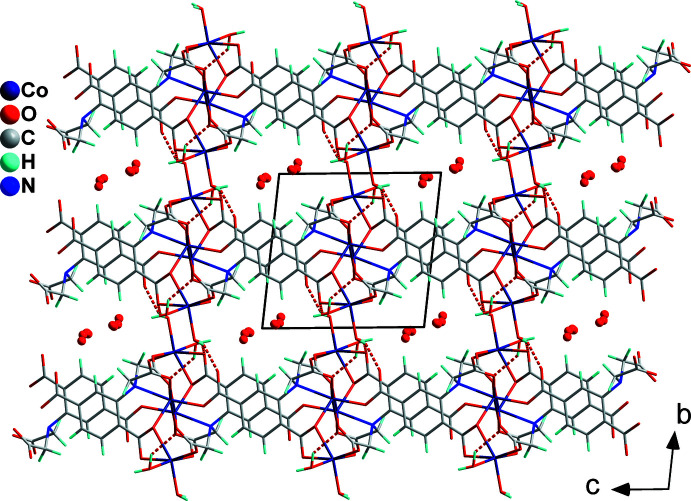
The three-dimensional supra­molecular network of (**I**) constructed *via* inter­molecular hydrogen bonds. The disordered water solvent mol­ecules are located in channels parallel to [100].

The (010) layers of (**I**) are assembled into a three-dimensional supra­molecular network *via* inter­molecular hydrogen bonds O9—H9*A*⋯O3^v^ and O9—H9*B*⋯O2^vi^ (Table 2 ▸, Fig. 4 ▸). The positionally and occupationally disordered solvent water mol­ecules (O11–O14) are situated in channels extending parallel to [100].

## Magnetic properties   

**Figure 5:**
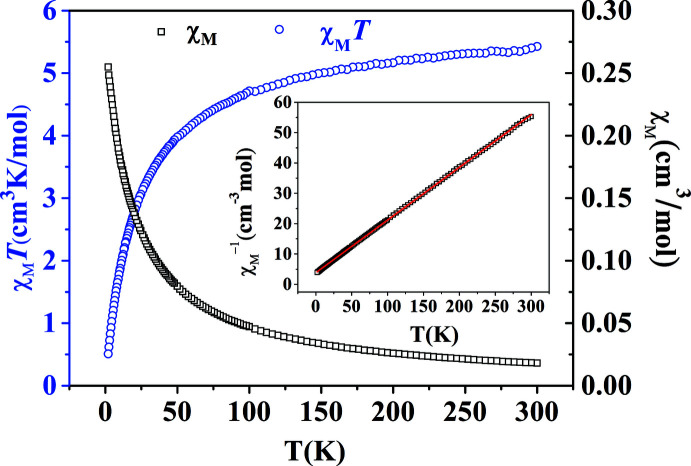
*χ*_M_ and *χ*_M_ *T versus. T* curves for compound (**I**). Inset: *χ*_M_^−1^ *versus T* plot. The red solid line represents the best-fit curve.

The variable-temperature magnetic susceptibilities (*χ*_M_) of (**I**) were measured in the range 2–300 K under 1000 Oe. The *χ*_M_, *χ*_M_^−1^ and *χ*_M_ *T* versus *T* plots are shown in Fig. 5 ▸. The value of *χ*_M_ *T* at 300 K is 5.43 cm^3^ K mol^−1^, which is much larger than the expected spin-only value (3.75 cm^3^ K mol^−1^) of two isolated Co^II^ ions with *g* = 2.0, *S* = 3/2, which may be due to the contribution of the incompletely quenched orbital magnetic moment. As the temperature decreases, the *χ*_M_ *T* value decreases slowly between 300 and 50 K and then it descends more steeply to the minimum value of 0.51 cm^3^ K mol^−1^ at 2 K. The curve clearly indicates that the dominant anti­ferromagnetic coupling is operating. The temperature dependence of *χ*_M_^−1^ follows the Curie–Weiss law, and the linear fit by the equation 1/*χ*_M_ = (*T* − *θ*)/*C* gives *C* = 5.76 cm^−3^ K mol^−1^ and *θ* = −21.99 K, which is consistent with an anti­ferromagnetic behaviour.

## Database survey   

A search of the Cambridge Structural Database (CSD version 5.42, May 2021 update; Groom *et al.*, 2016 ▸) for complexes with 2-aminodi­acetic terephthalic acid gave 19 hits, of which three are Co^II^ complexes including the title compound (Refcode: CUFDIS). The other two Co^II^ complexes are discrete coordination mol­ecules (Liu *et al.*, 2012 ▸). Three other complexes with layer structures based on 2-aminodi­acetic terephthalic acid without another organic ligand have also been reported, *viz*. MUMBON, an Mn^II^ complex (Ma *et al.*, 2015 ▸), NEVJIJ, a Cd^II^ complex (Ma *et al.*, 2013 ▸), and NEVJUV, a Zn^II^ complex (Ma *et al.*, 2013 ▸). NEVJUV has similar cell parameters to the title compound, but similar tetra­nuclear metal units are not found in NEVJUV because the Zn^II^ atoms have lower coordination numbers and the carboxyl­ate oxygen atoms do not bridge the Zn^II^ atoms as in the title compound. To the best of our knowledge, similar tetra­nuclear metal units have not been reported so far. Besides, one Co^II^ coordination polymer (CCDC reference: 2063370; Ma *et al.*, 2021 ▸), {[Co_2_(adtp)(H_2_O)_6_]·5H_2_O}_*n*_, has been synthesized, which consists of parallel stacked zigzag chains in which Co^II^ cations are linked together through μ_3_-adtp^4−^ anions.

## Synthesis and crystallization   

H_4_adtp was prepared using a similar protocol to that reported in the literature (Xu *et al.*, 2006 ▸). The other chemicals were purchased from commercial sources and used without further purification. A solution of 0.2 mmol (0.0594 g) H_4_adtp in 5.0 ml of H_2_O was adjusted to a pH of 6.1 by adding a 1.0 *M* KHCO_3_ solution drop by drop. The above solution was mixed with 0.5 mmol (0.1455 g) of Co(NO_3_)_2_·6H_2_O and 5.0 ml of CH_3_CN, then transferred into a 25.0 ml Teflon-lined stainless steel autoclave. The autoclave was sealed, heated to 393 K and held at that temperature for 72 h. The autoclave was allowed to cool to 303 K within 24 h. Plate-like pink crystals of (**I**) were collected in 66% yield based on H_4_adtp. Analysis calculated (%) for C_12_Co_2_N_1_O_11.6_H_14.2_ (*M*_r_ = 475.90): C 30.29, H 3.01, N, 2.94; found: C 30.18, H 3.15, N 3.06. Selected IR data (KBr pellet, cm^−1^): 3389 (*s*), 1631 (*s*), 1570 (*m*), 1405 (*s*), 1373 (*s*), 1319 (*b*), 1111 (*b*), 780 (*b*), 712 (*b*).

The phase purity of compound (**I**) was confirmed by powder X-ray diffraction analysis (PXRD; Fig. S1 in the supporting information). The peak positions of the experimental PXRD patterns are in good agreement with those simulated on basis of the present single-crystal X-ray data, indicating that a pure phase was obtained.

## Refinement   

Crystal data, data collection and structure refinement details are summarized in Table 3 ▸. The solvent water mol­ecules (O11, O12, O13 and O14) were found to be disordered and were refined isotropically with site occupancies of 0.5, 0.5, 0.35 and 0.25, respectively. The hydrogen atoms of the non-disordered water mol­ecules (O9, O10) were found in an difference density map and refined as riding, with *U*_iso_(H) = 1.5 *U*_eq_(O). Other hydrogen atoms were placed at geometrically calculated positions and treated as riding, with C*sp* ^2^—H = 0.93 Å, C*sp* ^3^—H = 0.97 Å and *U*_iso_(H) = 1.2 *U*_eq_(C). H atoms of O11, O12, O13 and O14 are not included in the model but were taken into account in the overall formula.

## Supplementary Material

Crystal structure: contains datablock(s) I. DOI: 10.1107/S2056989021008355/wm5615sup1.cif

Supporting information file. DOI: 10.1107/S2056989021008355/wm5615Isup3.cdx

Figure S1 The simulated and experimental PXRD patterns for compound (I). DOI: 10.1107/S2056989021008355/wm5615sup4.tif

CCDC reference: 2063394

Additional supporting information:  crystallographic information; 3D view; checkCIF report

## Figures and Tables

**Table 1:** Selected bond lengths (Å)

Co1—O5	2.049 (3)	Co2—O2^iii^	2.161 (2)
Co1—O7	2.033 (3)	Co2—O9	2.057 (3)
Co1—O7^i^	2.362 (3)	Co2—O8^iv^	2.065 (3)
Co1—O4^ii^	1.992 (3)	Co2—O6	2.038 (3)
Co1—O1	2.083 (3)	Co2—O1^iii^	2.212 (2)
Co1—N1	2.241 (3)	Co2—O10	2.126 (3)

Symmetry codes: (i) -x+1, -y+1, -z+1; (ii) x, y, z+1; (iii) -x, -y+1, -z+1; (iv) x-1, y, z.

**Table 2:** Hydrogen-bond geometry (Å, °)

*D*—H⋯*A*	*D*—H	H⋯*A*	*D*⋯*A*	*D*—H⋯*A*
O9—H9*A*⋯O3^v^	0.87	1.93	2.701 (4)	146
O9—H9*B*⋯O2^vi^	0.87	2.00	2.809 (4)	153
O10—H10*A*⋯O12	0.89	1.98	2.849 (10)	164
O10—H10*B*⋯O5	0.92	2.02	2.798 (4)	141

Symmetry codes: (v) -x, -y, -z; (vi) x-1, y-1, z.

**Table 3:** Experimental details

Crystal data	
Chemical formula	[Co_2_(C_12_H_7_NO_8_)(H_2_O)_2_]·1.6H_2_O
*M*_r_	475.90
Crystal system, space group	Triclinic, *P*\overline{1}
Temperature (K)	293
*a*, *b*, *c* (Å)	9.0064 (9), 9.2340 (8), 9.8426 (9)
α, β, γ (°)	93.859 (3), 105.571 (4), 99.483 (5)
*V* (Å^3^)	772.37 (13)
*Z*	2
Radiation type	Mo *K*α
μ (mm^−1^)	2.22
Crystal size (mm)	0.30 × 0.25 × 0.05

Data collection	
Diffractometer	Rigaku Saturn70 (4x4 bin mode)
Absorption correction	Multi-scan (*CrystalClear*; Rigaku, 2008 ▸)
*T*_min_, *T*_max_	0.908, 1.000
No. of measured, independent and observed [*I* > 2σ(*I*)] reflections	5012, 2619, 2245
*R*_int_	0.022
(sin θ/λ)_max_ (Å^−1^)	0.595

Refinement	
*R*[*F*^2^ > 2σ(*F* ^2^)], *wR*(*F* ^2^), *S*	0.035, 0.084, 1.04
No. of reflections	2619
No. of parameters	244
H-atom treatment	H-atom parameters constrained
Δρ_max_, Δρ_min_ (e Å^−3^)	0.80, −0.41

Computer programs: *CrystalClear* (Rigaku, 2008 ▸), *SHELXS* (Sheldrick, 2008 ▸), *SHELXL* (Sheldrick, 2015 ▸), *OLEX2* (Dolomanov *et al.*, 2009 ▸) and *publCIF* (Westrip, 2010 ▸).

## supplementary crystallographic information

### Crystal data

**Table:**

[Co_2_(C_12_H_7_NO_8_)(H_2_O)_2_]·(H_2_O)_1.6_	*Z* = 2
*M**_r_* = 475.90	*F*(000) = 480
Triclinic, *P*1	*D*_x_ = 2.046 Mg m^−^^3^
*a* = 9.0064 (9) Å	Mo *K*α radiation, λ = 0.71073 Å
*b* = 9.2340 (8) Å	Cell parameters from 1889 reflections
*c* = 9.8426 (9) Å	θ = 2.3–27.5°
α = 93.859 (3)°	µ = 2.22 mm^−^^1^
β = 105.571 (4)°	*T* = 293 K
γ = 99.483 (5)°	Plate, clear light red
*V* = 772.37 (13) Å^3^	0.30 × 0.25 × 0.05 mm

### Data collection

**Table:**

Rigaku Saturn70 (4x4 bin mode) diffractometer	2619 independent reflections
Radiation source: fine-focus sealed tube	2245 reflections with *I* > 2σ(*I*)
Graphite Monochromator monochromator	*R*_int_ = 0.022
Detector resolution: 28.5714 pixels mm^-1^	θ_max_ = 25.0°, θ_min_ = 3.0°
CCD_Profile_fitting scans	*h* = −10→10
Absorption correction: multi-scan (*CrystalClear*; Rigaku, 2008)	*k* = −10→10
*T*_min_ = 0.908, *T*_max_ = 1.000	*l* = −11→11
5012 measured reflections	

### Refinement

**Table:**

Refinement on *F*^2^	Primary atom site location: structure-invariant direct methods
Least-squares matrix: full	Hydrogen site location: mixed
*R*[*F*^2^ > 2σ(*F*^2^)] = 0.035	H-atom parameters constrained
*wR*(*F*^2^) = 0.084	*w* = 1/[σ^2^(*F*_o_^2^) + (0.035*P*)^2^ + 1.7231*P*] where *P* = (*F*_o_^2^ + 2*F*_c_^2^)/3
*S* = 1.04	(Δ/σ)_max_ < 0.001
2619 reflections	Δρ_max_ = 0.80 e Å^−^^3^
244 parameters	Δρ_min_ = −0.41 e Å^−^^3^
0 restraints	

### Special details

**Table:**

Geometry. All esds (except the esd in the dihedral angle between two l.s. planes) are estimated using the full covariance matrix. The cell esds are taken into account individually in the estimation of esds in distances, angles and torsion angles; correlations between esds in cell parameters are only used when they are defined by crystal symmetry. An approximate (isotropic) treatment of cell esds is used for estimating esds involving l.s. planes.

### Fractional atomic coordinates and isotropic or equivalent isotropic displacement parameters (Å^2^)

**Table:**

	*x*	*y*	*z*	*U*_iso_*/*U*_eq_	Occ. (<1)
Co1	0.29415 (6)	0.45959 (5)	0.48240 (5)	0.01675 (15)	
O5	0.0863 (3)	0.3198 (3)	0.4645 (3)	0.0269 (6)	
C1	0.2786 (4)	0.7012 (4)	0.3125 (4)	0.0166 (8)	
O7	0.4739 (3)	0.3593 (3)	0.4646 (3)	0.0194 (6)	
C2	0.2925 (4)	0.6185 (4)	0.1820 (4)	0.0163 (8)	
Co2	−0.25268 (6)	0.14862 (5)	0.47212 (5)	0.01611 (15)	
O2	0.3336 (3)	0.8376 (3)	0.3404 (3)	0.0213 (6)	
C3	0.3371 (5)	0.7042 (4)	0.0822 (4)	0.0209 (8)	
H3	0.362867	0.806555	0.103109	0.025*	
C4	0.3438 (5)	0.6402 (4)	−0.0464 (4)	0.0208 (8)	
H4	0.376198	0.698823	−0.110146	0.025*	
C5	0.3021 (4)	0.4882 (4)	−0.0798 (4)	0.0170 (8)	
C6	0.2603 (4)	0.4025 (4)	0.0199 (4)	0.0185 (8)	
H6	0.232662	0.300415	−0.002608	0.022*	
O9	−0.3388 (3)	−0.0735 (3)	0.4106 (3)	0.0273 (6)	
H9A	−0.294396	−0.105535	0.348644	0.041*	
H9B	−0.438376	−0.087425	0.364194	0.041*	
C7	0.2586 (4)	0.4641 (4)	0.1514 (4)	0.0166 (8)	
C8	0.2993 (4)	0.4154 (4)	−0.2221 (4)	0.0203 (8)	
O8	0.5342 (3)	0.1757 (3)	0.3424 (3)	0.0214 (6)	
C9	0.0396 (4)	0.3315 (4)	0.2165 (4)	0.0203 (8)	
H9C	−0.000542	0.253614	0.137501	0.024*	
H9D	−0.004209	0.417680	0.188115	0.024*	
C10	−0.0098 (4)	0.2809 (4)	0.3444 (4)	0.0194 (8)	
O4	0.3328 (3)	0.5005 (3)	−0.3085 (3)	0.0283 (7)	
C13	0.2808 (4)	0.2309 (4)	0.2585 (4)	0.0195 (8)	
H13A	0.285691	0.196842	0.164707	0.023*	
H13B	0.212473	0.154326	0.287781	0.023*	
C12	0.4434 (4)	0.2572 (4)	0.3616 (4)	0.0177 (8)	
O3	0.2624 (4)	0.2777 (3)	−0.2452 (3)	0.0328 (7)	
O6	−0.1453 (3)	0.2040 (3)	0.3200 (3)	0.0242 (6)	
O1	0.2057 (3)	0.6374 (3)	0.3941 (3)	0.0194 (6)	
O10	−0.0424 (3)	0.0957 (3)	0.6003 (3)	0.0286 (6)	
H10A	−0.052816	0.076051	0.685363	0.043*	
H10B	0.017174	0.187022	0.599944	0.043*	
N1	0.2142 (3)	0.3688 (3)	0.2523 (3)	0.0153 (6)	
O11	0.3252 (12)	0.0275 (10)	0.9277 (10)	0.069 (2)*	0.5
O12	−0.0171 (11)	0.0705 (10)	0.8922 (10)	0.074 (2)*	0.5
O13	0.2365 (15)	0.0205 (11)	0.9190 (11)	0.047 (3)*	0.35
O14	0.100 (3)	0.029 (3)	0.899 (3)	0.104 (7)*	0.25

### Atomic displacement parameters (Å^2^)

**Table:**

	*U*^11^	*U*^22^	*U*^33^	*U*^12^	*U*^13^	*U*^23^
Co1	0.0195 (3)	0.0195 (3)	0.0122 (3)	0.0038 (2)	0.0062 (2)	0.00157 (19)
O5	0.0250 (16)	0.0349 (16)	0.0172 (14)	−0.0055 (13)	0.0071 (12)	0.0012 (12)
C1	0.0154 (19)	0.0204 (19)	0.0136 (18)	0.0037 (15)	0.0040 (15)	0.0008 (14)
O7	0.0190 (14)	0.0198 (13)	0.0172 (13)	0.0035 (11)	0.0026 (11)	−0.0023 (10)
C2	0.016 (2)	0.0189 (18)	0.0141 (18)	0.0026 (15)	0.0051 (15)	0.0004 (14)
Co2	0.0175 (3)	0.0160 (3)	0.0165 (3)	0.0032 (2)	0.0082 (2)	0.00012 (19)
O2	0.0288 (16)	0.0168 (13)	0.0201 (14)	0.0024 (12)	0.0116 (12)	−0.0006 (10)
C3	0.027 (2)	0.0183 (18)	0.0181 (19)	0.0059 (16)	0.0073 (16)	0.0019 (15)
C4	0.024 (2)	0.025 (2)	0.0160 (19)	0.0061 (17)	0.0090 (16)	0.0060 (15)
C5	0.0161 (19)	0.0233 (19)	0.0116 (17)	0.0038 (16)	0.0043 (14)	0.0008 (14)
C6	0.023 (2)	0.0162 (17)	0.0161 (18)	0.0024 (15)	0.0063 (15)	0.0007 (14)
O9	0.0234 (16)	0.0212 (14)	0.0379 (17)	0.0016 (12)	0.0137 (13)	−0.0051 (12)
C7	0.016 (2)	0.0223 (19)	0.0122 (18)	0.0034 (16)	0.0061 (15)	0.0028 (14)
C8	0.021 (2)	0.028 (2)	0.0118 (18)	0.0054 (17)	0.0047 (15)	−0.0001 (15)
O8	0.0198 (14)	0.0211 (13)	0.0235 (14)	0.0074 (12)	0.0054 (11)	−0.0018 (11)
C9	0.014 (2)	0.029 (2)	0.0182 (19)	0.0028 (16)	0.0050 (15)	0.0048 (16)
C10	0.016 (2)	0.0204 (19)	0.023 (2)	0.0044 (16)	0.0073 (16)	0.0028 (15)
O4	0.0367 (18)	0.0335 (16)	0.0126 (13)	−0.0052 (13)	0.0117 (12)	−0.0010 (11)
C13	0.023 (2)	0.0166 (18)	0.0173 (19)	0.0030 (16)	0.0046 (16)	0.0001 (14)
C12	0.021 (2)	0.0157 (18)	0.0188 (19)	0.0019 (16)	0.0095 (16)	0.0052 (15)
O3	0.055 (2)	0.0272 (16)	0.0197 (15)	0.0098 (14)	0.0167 (14)	−0.0012 (12)
O6	0.0177 (15)	0.0345 (15)	0.0207 (14)	0.0010 (12)	0.0085 (11)	0.0043 (12)
O1	0.0217 (14)	0.0209 (13)	0.0174 (13)	0.0021 (11)	0.0107 (11)	−0.0001 (10)
O10	0.0263 (16)	0.0333 (16)	0.0275 (15)	0.0067 (13)	0.0076 (12)	0.0101 (12)
N1	0.0157 (16)	0.0177 (15)	0.0141 (15)	0.0030 (13)	0.0063 (12)	0.0037 (12)

### Geometric parameters (Å, º)

**Table:**

Co1—O5	2.049 (3)	C5—C8	1.504 (5)
Co1—O7	2.033 (3)	C6—H6	0.9300
Co1—O7^i^	2.362 (3)	C6—C7	1.383 (5)
Co1—O4^ii^	1.992 (3)	O9—H9A	0.8744
Co1—O1	2.083 (3)	O9—H9B	0.8742
Co1—N1	2.241 (3)	C7—N1	1.461 (4)
O5—C10	1.252 (5)	C8—O4	1.258 (5)
C1—C2	1.495 (5)	C8—O3	1.249 (5)
C1—O2	1.258 (4)	O8—C12	1.240 (4)
C1—O1	1.281 (4)	C9—H9C	0.9700
O7—C12	1.276 (4)	C9—H9D	0.9700
C2—C3	1.401 (5)	C9—C10	1.521 (5)
C2—C7	1.403 (5)	C9—N1	1.491 (5)
Co2—O2^iii^	2.161 (2)	C10—O6	1.259 (5)
Co2—O9	2.057 (3)	C13—H13A	0.9700
Co2—O8^iv^	2.065 (3)	C13—H13B	0.9700
Co2—O6	2.038 (3)	C13—C12	1.512 (5)
Co2—O1^iii^	2.212 (2)	C13—N1	1.492 (5)
Co2—O10	2.126 (3)	O10—H10A	0.8942
C3—H3	0.9300	O10—H10B	0.9225
C3—C4	1.381 (5)	O11—O13	0.771 (12)
C4—H4	0.9300	O12—O14	1.17 (3)
C4—C5	1.384 (5)	O13—O14	1.21 (3)
C5—C6	1.391 (5)		
			
O5—Co1—O7^i^	170.67 (10)	C6—C5—C8	119.9 (3)
O5—Co1—O1	99.02 (11)	C5—C6—H6	118.9
O5—Co1—N1	76.91 (11)	C7—C6—C5	122.2 (3)
O7—Co1—O5	115.34 (11)	C7—C6—H6	118.9
O7—Co1—O7^i^	71.31 (11)	Co2—O9—H9A	109.7
O7—Co1—O1	133.46 (10)	Co2—O9—H9B	109.8
O7—Co1—N1	78.36 (10)	H9A—O9—H9B	104.3
O4^ii^—Co1—O5	91.10 (11)	C2—C7—N1	121.3 (3)
O4^ii^—Co1—O7^i^	80.73 (10)	C6—C7—C2	118.7 (3)
O4^ii^—Co1—O7	103.42 (11)	C6—C7—N1	119.9 (3)
O4^ii^—Co1—O1	106.43 (11)	O4—C8—C5	116.2 (3)
O4^ii^—Co1—N1	167.23 (11)	O3—C8—C5	118.0 (3)
O1—Co1—O7^i^	79.19 (9)	O3—C8—O4	125.8 (3)
O1—Co1—N1	80.14 (10)	C12—O8—Co2^v^	130.6 (2)
N1—Co1—O7^i^	111.56 (10)	H9C—C9—H9D	108.2
C10—O5—Co1	119.4 (2)	C10—C9—H9C	109.6
O2—C1—C2	119.7 (3)	C10—C9—H9D	109.6
O2—C1—O1	118.9 (3)	N1—C9—H9C	109.6
O1—C1—C2	121.4 (3)	N1—C9—H9D	109.6
Co1—O7—Co1^i^	108.69 (10)	N1—C9—C10	110.1 (3)
C12—O7—Co1^i^	120.4 (2)	O5—C10—C9	117.6 (3)
C12—O7—Co1	116.8 (2)	O5—C10—O6	125.6 (3)
C3—C2—C1	116.4 (3)	O6—C10—C9	116.8 (3)
C3—C2—C7	118.8 (3)	C8—O4—Co1^vi^	129.3 (2)
C7—C2—C1	124.8 (3)	H13A—C13—H13B	108.0
O2^iii^—Co2—O1^iii^	59.97 (9)	C12—C13—H13A	109.4
O9—Co2—O2^iii^	96.68 (10)	C12—C13—H13B	109.4
O9—Co2—O8^iv^	84.76 (11)	N1—C13—H13A	109.4
O9—Co2—O1^iii^	156.66 (10)	N1—C13—H13B	109.4
O9—Co2—O10	89.06 (11)	N1—C13—C12	111.1 (3)
O8^iv^—Co2—O2^iii^	92.24 (10)	O7—C12—C13	116.7 (3)
O8^iv^—Co2—O1^iii^	95.29 (10)	O8—C12—O7	125.3 (3)
O8^iv^—Co2—O10	173.82 (11)	O8—C12—C13	117.9 (3)
O6—Co2—O2^iii^	161.71 (11)	C10—O6—Co2	124.7 (2)
O6—Co2—O9	101.55 (11)	Co1—O1—Co2^iii^	119.35 (12)
O6—Co2—O8^iv^	90.90 (11)	C1—O1—Co1	115.4 (2)
O6—Co2—O1^iii^	101.79 (10)	C1—O1—Co2^iii^	89.1 (2)
O6—Co2—O10	90.63 (11)	Co2—O10—H10A	110.5
O10—Co2—O2^iii^	88.17 (11)	Co2—O10—H10B	93.8
O10—Co2—O1^iii^	90.26 (10)	H10A—O10—H10B	114.8
C1—O2—Co2^iii^	92.0 (2)	C7—N1—Co1	117.5 (2)
C2—C3—H3	119.3	C7—N1—C9	109.1 (3)
C4—C3—C2	121.5 (3)	C7—N1—C13	113.8 (3)
C4—C3—H3	119.3	C9—N1—Co1	105.0 (2)
C3—C4—H4	120.2	C9—N1—C13	110.2 (3)
C3—C4—C5	119.7 (3)	C13—N1—Co1	100.6 (2)
C5—C4—H4	120.2	O11—O13—O14	171 (2)
C4—C5—C6	119.0 (3)	O12—O14—O13	164 (2)
C4—C5—C8	121.1 (3)		
			
Co1—O5—C10—C9	4.5 (4)	C4—C5—C8—O4	−1.8 (5)
Co1—O5—C10—O6	−174.9 (3)	C4—C5—C8—O3	179.1 (4)
Co1^i^—O7—C12—O8	51.6 (4)	C5—C6—C7—C2	3.3 (6)
Co1—O7—C12—O8	−173.0 (3)	C5—C6—C7—N1	−179.8 (3)
Co1—O7—C12—C13	4.5 (4)	C5—C8—O4—Co1^vi^	−163.4 (2)
Co1^i^—O7—C12—C13	−130.8 (3)	C6—C5—C8—O4	177.4 (4)
O5—C10—O6—Co2	9.2 (5)	C6—C5—C8—O3	−1.7 (5)
C1—C2—C3—C4	−175.7 (3)	C6—C7—N1—Co1	158.5 (3)
C1—C2—C7—C6	173.2 (3)	C6—C7—N1—C9	−82.2 (4)
C1—C2—C7—N1	−3.7 (6)	C6—C7—N1—C13	41.4 (5)
C2—C1—O2—Co2^iii^	178.1 (3)	C7—C2—C3—C4	1.9 (6)
C2—C1—O1—Co1	59.6 (4)	C8—C5—C6—C7	−179.0 (3)
C2—C1—O1—Co2^iii^	−178.0 (3)	C9—C10—O6—Co2	−170.2 (2)
C2—C3—C4—C5	1.6 (6)	C10—C9—N1—Co1	−33.2 (3)
C2—C7—N1—Co1	−24.7 (4)	C10—C9—N1—C7	−160.1 (3)
C2—C7—N1—C9	94.7 (4)	C10—C9—N1—C13	74.3 (4)
C2—C7—N1—C13	−141.8 (3)	C12—C13—N1—Co1	−40.3 (3)
Co2^v^—O8—C12—O7	9.3 (5)	C12—C13—N1—C7	86.3 (4)
Co2^v^—O8—C12—C13	−168.3 (2)	C12—C13—N1—C9	−150.7 (3)
O2—C1—C2—C3	−13.0 (5)	O3—C8—O4—Co1^vi^	15.5 (6)
O2—C1—C2—C7	169.5 (3)	O1—C1—C2—C3	164.0 (3)
O2—C1—O1—Co1	−123.4 (3)	O1—C1—C2—C7	−13.6 (6)
O2—C1—O1—Co2^iii^	−1.0 (3)	O1—C1—O2—Co2^iii^	1.1 (3)
C3—C2—C7—C6	−4.3 (5)	N1—C9—C10—O5	22.0 (5)
C3—C2—C7—N1	178.9 (3)	N1—C9—C10—O6	−158.6 (3)
C3—C4—C5—C6	−2.6 (6)	N1—C13—C12—O7	27.9 (4)
C3—C4—C5—C8	176.6 (3)	N1—C13—C12—O8	−154.3 (3)
C4—C5—C6—C7	0.2 (6)		

Symmetry codes: (i) −*x*+1, −*y*+1, −*z*+1; (ii) *x*, *y*, *z*+1; (iii) −*x*, −*y*+1, −*z*+1; (iv) *x*−1, *y*, *z*; (v) *x*+1, *y*, *z*; (vi) *x*, *y*, *z*−1.

### Hydrogen-bond geometry (Å, º)

**Table:**

*D*—H···*A*	*D*—H	H···*A*	*D*···*A*	*D*—H···*A*
O9—H9*A*···O3^vii^	0.87	1.93	2.701 (4)	146
O9—H9*B*···O2^viii^	0.87	2.00	2.809 (4)	153
O10—H10*A*···O12	0.89	1.98	2.849 (10)	164
O10—H10*B*···O5	0.92	2.02	2.798 (4)	141

Symmetry codes: (vii) −*x*, −*y*, −*z*; (viii) *x*−1, *y*−1, *z*.
